# Effect of ambient air pollutants and meteorological variables on COVID-19 incidence

**DOI:** 10.1017/ice.2020.222

**Published:** 2020-05-11

**Authors:** Ying Jiang, Xiao-Jun Wu, Yan-Jun Guan

**Affiliations:** 1 Department of Neurosurgery, Shanghai Chang Zheng Hospital affiliated with China Second Military Medical University, Shanghai, China; 2 Department of Neurosurgery, Shanghai Cancer Center, Shanghai Fu-Dan University School of Medicine, Shanghai, China; 3 Department of Otorhinolaryngology, Shanghai Rui-Jin Hospital, Shanghai Jiaotong University School of Medicine, Shanghai, China

## Abstract

**Objective:**

To determine whether ambient air pollutants and meteorological variables are associated with daily COVID-19 incidence.

**Design:**

A retrospective cohort from January 25 to February 29, 2020.

**Setting:**

Cities of Wuhan, Xiaogan, and Huanggang, China.

**Patients:**

The COVID-19 cases detected each day.

**Methods:**

We collected daily data of COVID-19 incidence, 8 ambient air pollutants (particulate matter of ≤2.5 µm [PM_2.5_], particulate matter ≤10 µm [PM_10_], sulfur dioxide [SO_2_], carbon monoxide [CO], nitrogen dioxide [NO_2_], and maximum 8-h moving average concentrations for ozone [O_3_-8h]) and 3 meteorological variables (temperature, relative humidity, and wind) in China’s 3 worst COVID-19–stricken cities during the study period. The multivariate Poisson regression was performed to understand their correlation.

**Results:**

Daily COVID-19 incidence was positively associated with PM_2.5_ and humidity in all cities. Specifically, the relative risk (RR) of PM_2.5_ for daily COVID-19 incidences were 1.036 (95% confidence interval [CI], 1.032–1.039) in Wuhan, 1.059 (95% CI, 1.046–1.072) in Xiaogan, and 1.144 (95% CI, 1.12–1.169) in Huanggang. The RR of humidity for daily COVID-19 incidence was consistently lower than that of PM_2.5_, and this difference ranged from 0.027 to 0.111. Moreover, PM_10_ and temperature also exhibited a notable correlation with daily COVID-19 incidence, but in a negative pattern The RR of PM_10_ for daily COVID-19 incidence ranged from 0.915 (95% CI, 0.896–0.934) to 0.961 (95% CI, 0.95–0.972, while that of temperature ranged from 0.738 (95% CI, 0.717–0.759) to 0.969 (95% CI, 0.966–0.973).

**Conclusions:**

Our data show that PM_2.5_ and humidity are substantially associated with an increased risk of COVID-19 and that PM_10_ and temperature are substantially associated with a decreased risk of COVID-19.

Since it first appeared in Wuhan, China, in December 2019, coronavirus disease 2019 (COVID-19) has become a worldwide pandemic.^1–3^ Severe acute respiratory syndrome coronavirus-2 (SARS-CoV-2) is the pathogen of the COVID-19, and it now poses a threat to global health. As of March 31, 2020, 785,979 confirmed cases and 37,810 deaths related to COVID-19 had been recorded among 204 countries.^4^

Ambient air pollution is a well-known threat to human health, and sufficient evidence is available to support the close correlation between pollutants and increased risks of numerous diseases.^5–7^ In particular, ambient air pollutants have raised concerns over their association with infectious diseases, some of which have caused local epidemics.^8–10^ It has been speculated that airborne pollutants provide “condensation nuclei” to which virus particles can attach.^11^ This hypothesis is supported by the influenza–PM_2.5_ correlation.^10,12,13^ Although SARS-CoV-2 is known to be transmitted human to human by infectious secretions,^3^ these secretions can be transferred in many different ways. Thus, whether ambient air pollutants could affect the transmission of SARS-CoV-2 is an urgent question. Additionally, meteorological factors such as humidity and temperature have also been suggested to enhance air pollution^14,15^ and to promote transmission of infectious disease.^14^ Their role in SARS-CoV-2 transmission, however, remains largely unknown.

Wuhan is one of the most populated cities in China, and it has a subtropical humid monsoon climate. Due to rapid industrialization and urbanization, both the concentration and composition of air pollutants have become higher and more complicated,^16,17^ and pollution has already caused severe health problems for the local population.^18^ A previous study demonstrated that a tuberculosis surge in Wuhan was associated with worsening air pollution and weather.^19^ Nevertheless, no study has explored the association of Wuhan local air quality and/or meteorological data with COVID-19 incidence.

Due to the severe air pollution, high COVID-19, and the citywide lockdown, Wuhan is a good research setting in which to investigate this issue. We hypothesized the potential association of air pollutants and meteorological variables with daily COVID-19 incidence, and we sought to discover factors influencing the SARS-CoV-2 pandemic. We also enrolled Xiaogan and Huanggang in the current study because they were the second- and third-worst COVID-19–stricken cities in the province of Wuhan, respectively.

## Methods

### Data sources

#### Ambient air pollutants and meteorological variables

The daily air quality index (AQI) and average concentration data for 6 ambient air pollutants data were collected from the AQI platform website (https://www.aqistudy.cn). These pollutants included particulate matter of ≤2.5 µm (PM_2.5_), particulate matter ≤10 µm (PM_10_), sulfur dioxide (SO_2_]), carbon monoxide (CO), nitrogen dioxide (NO_2_), and maximum 8-h moving average concentrations for ozone (O_3_-8h). We calculated their daily concentrations by averaging the hourly concentrations from all stations in the city to represent citywide pollution exposure. The website uses the standard for particulate matter from the US Environmental Protection Agency (EPA).^20^ The daily temperature, relative humidity, and wind (in Beaufort number) were also obtained from the AQI platform, and we averaged the hourly data from all stations in the city to represent the citywide weather condition.

#### Population

The study population included a retrospective cohort of COVID-19 patients in Wuhan, Xiaogan and Huanggang, China, between January 25 and February 29, 2020. The population of each city was obtained from the China National Bureau of Statistics. Because all 3 cities were under a citywide lockdown during the entire study period, the population size was considered stable.

#### COVID-19 diagnosis and daily incidence

The daily COVID-19 incidence was obtained from the website of Health Commission of Hubei Province from January 25, 2020, onward.^21^ The COVID-19 diagnoses were made solely based on nucleic acid test from January 25 to February 11. Beginning February 12, clinical symptoms along with chest computed tomography (CT) scans were used for COVID-19 diagnoses.^22,23^ When COVID-19 was diagnosed, the patient was immediately placed in a single isolation ward. These remained in quarantine until they fully recovered or died. A previous epidemiological study demonstrated that the median incubation period of SARS-CoV-2 is 4 days.^24^ Thus, we applied this correlation to analyze the relationship between COVID-19 incidence per day and the air pollution data 4 days prior.

### Data analysis

We conducted a time-series analysis to examine the associations of the pollutants and meteorological variables with COVID-19 incidence per day. We first performed a descriptive analysis that provided the information for COVID-19 incidence, 6 air pollutants, and 3 meteorological variables in all 3 cities. We then used multivariate Poisson regression models to evaluate the association of pollutants and meteorological variables with COVID-19 incidence in 3 cities on a daily basis. All analyses were conducted using SPSS version 20.0 software (SPSS, Chicago, IL). Bonferroni correction was used for data correction, and a *P* value <.0056 was considered as statistically significant.

## Results

Table 1 lists the descriptive statistics for daily COVID-19 incidence, air pollutant concentration, and meteorological variables in Wuhan, Xiaogan, and Huanggang. Starting January 26, Wuhan had 80 newly diagnosed COVID-19 cases, Xiaogan had 45, and Huanggang had 32. All cities reached their peak daily incidence between February 12 and 13. Thereafter, the daily incidences started to drop, and they reached their lowest by the end of the study.

**Table 1.** Descriptive statistics of coronavirus disease 2019 (COVID-19) incidences per day, three meteorological variables and six ambient air pollutants conditions from 25^th^ Jan to 29^th^ Feb 2020 in Wuhan, XiaoGan and HuangGang, China (total 36 days). Min., Max., and SEM stand for minimum, maximum and standard error of mean, respectively

	Wuhan			XiaoGan			HuangGang		
	Min.	Max.	Mean (SEM)	Min.	Max.	Mean (SEM)	Min.	Max.	Mean (SEM)
**Incidence (cases)**	80	13436	1386 ± 373.5	−15	424	98.94 ± 16.17	−5	276	79.51 ± 14.45
**Meteorological Variables**									
Temperature (ºC)	2	18	7.11 ± 0.67	2	17	6.86 ± 0.63	3	18	7.22 ± 0.63
Relative Humidity (%)	58	95	79.33 ± 1.47	58	98	81.69 ± 1.96	53	98	79 ± 2.29
Wind (Beaufort No.)	0	3	0.75 ± 0.14	1	3	1.17 ± 0.07	1	4	1.47 ± 0.11
**Ambient Air Pollutants (μg/m**^**3**^**)**									
PM_2.5_	11	97	45.53 ± 3.84	12	108	50.28 ± 4.27	16	85	46.08 ± 3.55
PM_10_	20	103	53.08 ± 3.99	22	122	59.58 ± 4.69	30	114	67.06 ± 3.87
SO_2_	5	13	7.36 ± 0.38	4	14	7.03 ± 0.44	4	14	6.83 ± 0.39
CO	0.5	1.3	0.89 ± 0.03	0.8	1.6	1.15 ± 0.04	0.6	1.3	0.87 ± 0.03
NO_2_	10	47	22.25 ± 1.42	4	26	11.61 ± 0.81	8	24	15.25 ± 0.76
O_3_-8h	45	110	75.39 ± 3.64	40	120	78.72 ± 3.7	33	112	75.28 ± 3.81

Next, multiple linear relationships were used to evaluate the association of the daily COVID-19 incidence with air pollutants and meteorological variables in each city (Table 2). Due to the change in case definition on February 12, the COVID-19 incidence surged on February 12 and 13.^22,23^ Additionally, Xiaogan and Huanggang showed negative COVID-19 incidences on February 17 and 19. Thus, we excluded data from these dates in our multiple linear regression analysis. First, in Wuhan (Table 2), all air pollutants (except SO_2_) were strongly associated with daily COVID-19 incidence (all *P* < .001). Among them, PM_10_ exhibited a negative association with COVID-19 incidence (relative risk [RR], 0.964; 95% confidence interval [CI], 0.961–0.967). Among the remaining pollutants, PM_2.5_ was a main effector on incidence increase (RR, 1.036; 95% CI, 1.032–1.039). Among the 3 meteorological variables, temperature (RR, 0.969; 95% CI, 0.966–0.973) and humidity (RR, 1.009; 95% CI, 1.007–1.011) were significantly associated with daily COVID-19 incidence (both *P* < .001).

**Table 2.** The association between the coronavirus disease 2019 (COVID-19) incidence per day and six ambient air pollutants/three meteorological variables from 25^th^ Jan to 29^th^ Feb 2020 in Wuhan, XiaoGan and HuangGang, China (total 35 days). RR and 95% CI stand for relative ratio and 95% confidence interval, respectively. The number marked with # indicated association between factors and COVID-19 incidence were NOT statistically significant (P>0.0056). Meanwhile, the p-value of those not marked data were <0.01 and considered statistically significant

	Wuhan			XiaoGan			HuangGang		
	Regression Coefficient	RR	95% CI	Regression Coefficient	RR	95% CI	Regression Coefficient	RR	95% CI
**Incidence (cases)**									
**Meteorological Variables**									
Temperature (ºC)	−0.031	0.969	0.966–0.973	−0.116	0.89	0.871–0.911	−0.304	0.738	0.717–0.759
Relative Humidity (%)	0.009	1.009	1.007–1.011	0.013	1.013	1.007–1.019	0.032	1.033	1.026–1.039
Wind (Beaufort No.)	−0.011 ^#^	0.989	0.973–1.006	−0.287	0.75	0.655–0.859	−0.236	0.79	0.697–0.896
**Ambient Air Pollutants (μg/m**^**3**^**)**									
PM_2.5_	0.035	1.036	1.032–1.039	0.057	1.059	1.046–1.072	0.135	1.144	1.12 – 1.169
PM_10_	−0.037	0.964	0.961–0.967	−0.04	0.961	0.95–0.972	−0.089	0.915	0.896–0.934
SO_2_	0.002^#^	1.002	0.990–1.014	0.182	1.199	1.166–1.233	0.05	1.051	1.018–1.085
CO	0.659	1.932	1.763–2.118	−3.186	0.041	0.026–0.066	−3.427	0.032	0.017–0.063
NO_2_	0.054	1.056	1.053–1.059	0.109	1.115	1.095–1.136	0.004 ^#^	1.004	0.981–1.028
O_3_-8h	−0.01	0.99	0.989–0.991	−0.009	0.991	0.989–0.993	0.016	1.016	1.012–1.02

In XiaoGan, all air pollutants were notably associated with daily COVID-19 incidence (all *P* < .001). Similar to that of Wuhan, both PM_10_ (RR, 0.961; 95% CI, 0.95–0.972) and temperature (RR, 0.89; 95% CI, 0.871–0.911) remained negatively correlated with daily COVID-19 incidence. Among the remaining air pollutants, COVID-19 incidence increase was also significantly associated with PM_2.5_ (RR, 1.059; 95% CI, 1.046–1.072). For 3 meteorological variables, humidity (RR, 1.013; 95% CI, 1.007–1.019) and temperature (RR, 0.89; 95% CI, 0.871–0.911) were the 2 main factors associated with COVID-19 incidence.

In Huanggang, all ambient air pollutants (expect NO_2_) were significantly associated with daily COVID-19 incidence (all *P* < .001). Similar to Wuhan and Xiaogan, PM_10_ remained negatively correlated with the daily COVID-19 incidence (RR, 0.915; 95% CI, 0.896–0.934). Among the remaining air pollutants, PM_2.5_ had the strongest correlation with COVID-19 incidence (RR, 1.144; 95% CI, 1.12–1.169). All meteorological variables were also significantly associated with daily COVID-19 incidence (both *P* < .001). Similar to the previous 2 cities, a negative association was detected between COVID-19 and temperature (RR, 0.738; 95% CI, 0.717–0.759) and between COVID-19 and wind level (RR, 0.79; 95% CI, 0.697–0.896).

## Discussion

Ambient air pollution and meteorological variables can affect viral transmission; however, they have not yet been examined for the presence of SARS-CoV-2. In the current study, we have provided an initial assessment of the potential effects of ambient air pollutants and meteorological variables on the incidence of COVID-19 day by day from January 25 to February 29, 2020. Overall, based on data from China’s 3 worst COVID-19–stricken cities, COVID-19 incidence was negatively associated with PM_10_ and temperature. Meanwhile, a positive association was observed between COVID-19 and PM_2.5_ and relative humidity.

Particulate matter is a mixture of both solid particles and liquid droplets suspended in the air. Although it is not the sole cause of respiratory illness, previous studies have demonstrated that particulate matter is a strong environmental determinant of viral transmissions. For example, a close correlation was discovered between human influenza cases and PM_2.5_ concentrations in epidemiological studies based in big cities such as Beijing,^12^ Hong Kong,^25^ and Brisbane.^26^ Its potential mechanisms are believed to damage bronchial immunity^27^ and epithelial cell integrity,^28^ which enhances viral attachment to and replication in the bronchus.^28^ In the current study, although our data could not reveal whether particulate matter promotes SARS-CoV-2 transmission, we observed that PM_2.5_ is the only air pollutant that is consistently associated with increased COVID-19 incidence in all cities. This finding agrees with the data from a previous influenza study. Contrary to PM_2.5_, increased PM_10_ concentration was associated with decreased COVID-19 incidence. We believe this phenomenon to be a diameter-related effect. As shown by previous study, the receptor for both SARS-CoV-2 and SARS-CoV binding is the angiotensin-converting enzyme 2, which concentrates on type II alveolar cells.^29^ However, type II alveolar cells are located in the alveoli, which can only be reached by particles with a diameter <5 µm.^30^ Thus, small airborne pollutants, such as PM_2.5_, are particularly harmful because they are more likely to penetrate the respiratory tract all the way to the alveolar region unfiltered.^31,32^ For larger particulate matter, penetration ability decreases dramatically when size increases; starting at 20 µm and beyond, particulate matter is not able to penetrate below the trachea.^32^ Although all particulate matter might provide condensation nuclei for viral attachment,^11^ PM_2.5_ deliver SARS-CoV-2 to their target cells in the alveoli, which is unreachable to PM_10_. This size factor might also explain our finding that PM_2.5_ correlates with COVID-19 incidence in a positive pattern while that of PM_10_ is negative. Future epidemiological studies in other countries and areas with similar population density but different PM_2.5_ and PM_10_ concentrations could be used to validate this result.

So far, a number of studies have indicated a negative effect of ambient temperature and humidity on the viral viability and transmission. For example, low humidity increases influenza viability in the aerosol^30^ and impairs the innate antiviral defense of the host,^33^ which promotes influenza transmission. Meanwhile, temperature has the same effect as humidity on influenza.^34^ Similar to influenza, epidemiological studies have also demonstrated that the transmission of SARS-CoV-2 is more efficient in low temperatures and at low humidity.^35,36^ This hypothesis was supported by the results from a laboratory study; SARS-CoV was more likely to be inactivated in higher temperature and humidity.^37,38^ In the current study, our data agree with previous findings that temperature is negatively associated with COVID-19 incidence. However, our results regarding humidity do not agree with those from previous studies. Recently, an outbreak of COVID-19 was reported in a public bath center in China.^39^ Since the bath center was extremely humid, SARS-CoV-2 should have been inactivated quickly; however, this was not the case. Thus, our results support this finding and suggest that the study of differences between SARS-CoV-2 and other viruses is warranted in future studies.

Whether SARS-CoV-2 can be transmitted via airborne droplets remains controversial.^40^ Recently, Luo et al^39^ reported a COVID-19 outbreak in a public bath center in China, which suggests the role of ambient droplet in SARS-CoV-2 transmission in conditions of high relative humidity. Later, van Doremalen et al^41^ also demonstrated that, under laboratory conditions, SARS-CoV-2 is viable in aerosol droplets for at least 3 hours, which provides theoretical evidence for how SARS-CoV-2 transmission in that environment. However, some researchers dispute ambient droplet transmission because SARS-CoV-2 is not present in air samples obtained from the rooms of hospitalized COVID-19 patients.^41^ Most studies thus far have also reported that healthy people are infected by touching contaminated objects^40^ and that SARS-CoV-2 is stable on copper, cardboard, plastic, and stainless-steel surfaces for up to 72 hours.^41^ In the current study, we have shown that ambient air pollutants, especially PM_2.5_, are closely associated with COVID-19 incidence, which supports the hypothesis regarding airborne droplet transmission of SARS-CoV-2. However, the viability of SARS-CoV-2 attaching to PM_2.5_ remains largely unknown. Further studies are required to investigate the extent of SARS-CoV-2 transmission via airborne droplets.

Winter features overlap multiple viral seasons at various degrees. Previous studies have suggested that epidemics of cocirculating viral infections can interfere with each other.^42,43^ For example, influenza A could overlap coronavirus outbreaks.^42^ Thus, it is reasonable to hypothesize that COVID-19 incidence might also be significantly affected by other cocirculating viruses. Additionally, 1 study concluded that 2 SARS-CoV-2 strains are cocirculating in Italy.^44^ Whether these 2 strains affect the infectious ability of the other remains unknown. Further study is required to fully elucidate this issue.

The current study has several limitations. First, because the ambient PM_2.5_ is a mixture of solid particles and liquid droplets, the exact components of PM_2.5_ that may promote coronavirus transmission remain unknown. Second, due to the relatively short study period and imperfect daily reporting practices, our results are vulnerable to changes resulting from the emergence of more detailed data. Third, we studied only 9 variables in the current study. A number of other variables could potentially have affected COVID-19 transmission; thus, our study can only provide preliminary information on the association between COVID-19 and air pollutants or other meteorological variables. In a future study, we plan to gather data from more countries and regions to remedy these data gaps. Fourth, because detailed information from confirmed COVID-19 patients was not available, we were unable to determine how underlying health problems might promote COVID-19 infection. Thus, a longer study period might have affected our results. Moreover, winter overlapped the entire study period, which limited all meteorological variables (especially temperature) to a narrow band. Thus, further study is urgently needed further elucidate these issues.

In conclusion, our findings consistently suggest that increased temperature and airborne PM_2.5_ concentration are associated with increased daily COVID-19 incidence. Meanwhile, a negative association was observed between COVID-19 incidence and relative humidity and airborne PM_10_ concentration. In the context of the worldwide COVID-19 pandemic, our findings might provide some information for COVID-19 personal protection and transmission reduction.
